# Repeatable detection of Ag^+^ ions using a DNA aptamer-linked hydrogel biochemical sensor integrated with microfluidic heating system

**DOI:** 10.1038/s41598-022-13970-z

**Published:** 2022-06-11

**Authors:** Koki Yoshida, Tomoki Hayashi, Masahiro Takinoue, Hiroaki Onoe

**Affiliations:** 1 Graduate School of Integrated Design Engineering, Keio University, 3-14-1 Hiyoshi, Kohoku-Ku, Yokohama, 223-8522 Japan; 2 Department of Mechanical Engineering, Faculty of Science and Technology, Keio University, 3-14-1 Hiyoshi, Kohoku-Ku, Yokohama, 223-8522 Japan; 3 Department of Computer Science, School of Computing, Tokyo Institute of Technology, 4259 Nagatsutacho, Midori-Ku, Yokohama, 226-8502 Japan

**Keywords:** Chemical engineering, Mechanical engineering

## Abstract

This paper describes repeatable detection of Ag^+^ ions using a DNA aptamer-linked hydrogel biochemical sensor integrated with a microfluidic heating system. Biochemical sensors that respond to chemical compounds and produce detectable signals have a critical role in many aspects of modern society. In particular, the repeatable measurement of environmental information such as toxic substances including Ag^+^ ions could be expected to improve the environment. The DNA aptamer is an attractive candidate because of the stability and the selectivity of binding to chemicals. However, previous DNA aptamer biochemical sensors could not measure repeatedly because those sensors did not have initializing functions. To overcome this challenge, we proposed a DNA aptamer-linked hydrogel biochemical sensor integrated with the microfluidic heating system enabling repeatable detection of Ag^+^ ions. The binding Ag^+^ ions are dissociated by heating and flushing through the integrated microfluidic heating device. The DNA aptamer-linked hydrogel had the capability to detect a wide range of Ag^+^ ion concentrations (10^−5^–10 mM) including a toxic range for various aquatic organisms. Finally, we demonstrated the repeatable detection of the Ag^+^ ions. These results indicated that our proposed biochemical sensor is expected to use for long-term monitoring with high stability in ambient temperature and low power consumption.

## Introduction

Biochemical sensors that respond to chemical compounds and produce detectable signals has a critical role in many aspects of modern society because human are much less sensitive to the chemical or biological environment than to the physical environment such as light, pressure, temperature, or humidity^1^. For example, the specific molecules inside a human body such as metabolites, metal ions, and hormones reflect the person’s health, while chemicals in the environment including heavy metals, explosives, and toxins can influence plants and animals including humans^2,3^. For promoting a person's health and improving the environment, it is highly expected to measure such biometric and environmental information repeatedly over a long period. Toward the realization of repeatable measurement for the long period, the development of highly sensitive and repeatable biochemical sensors is anticipated.

Although various types of biochemical sensors have been developed^4–7^, functional nucleic acid sensors^1^, that use DNA, RNA, or other nucleic acids as a sensing element, have recently been focused on as new-generation biochemical sensors. Among the functional nucleic acid sensors, DNA aptamer, that is single-stranded DNA with a specific base sequence, has attracted much attention because a DNA aptamer binds specifically to a target substance with high binding selectivity. Up to date, various target substances for DNA aptamers have been reported including biological substances such as platelet-derived growth factor, thrombin, cocaine, and environmentally toxic substances including Ag^+^ ions, Hg^2+^ ions, Pb^2+^ ions, and Sr^2+^ ions^8,9^. In addition, DNA aptamers are stable materials: The DNA aptamer could be thermal denatured and renatured for many cycles without losing binding ability^1^. Taking advantage of these characteristics, various types of DNA aptamer biochemical sensors have been developed^9,10^. Most of these DNA aptamer biochemical sensors employ fluorescence^11,12^, electrochemical^13,14^, and visible wavelength^15^ signals as their outputs. However, those DNA aptamer biochemical sensors could measure their target molecules only once because those sensors did not have a function to dissociate the binding of the target molecules to the DNA aptamer. For repeatable measurement of the DNA aptamer biochemical sensor, it is necessary to initialize the DNA aptamer biochemical sensor by dissociating and removing the target molecule in the DNA aptamer biochemical sensors.

**Figure 1:**
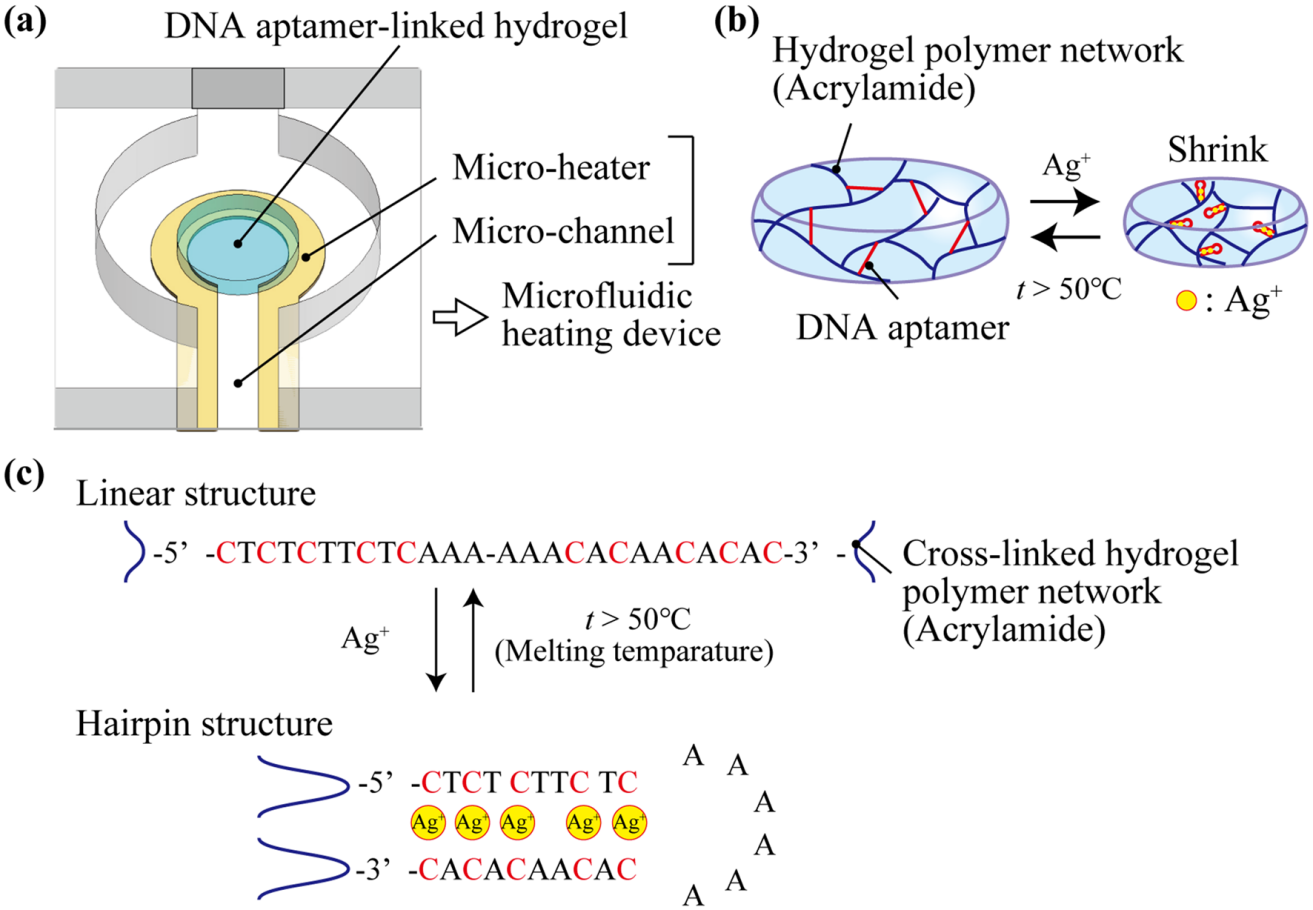
(**a**) Schematic illustration of the DNA aptamer-linked hydrogel biochemical sensor integrated with the microfluidic heating device for repeatable detection of chemical substances. (**b**) The DNA aptamer-linked hydrogel changes its volume caused by forming the hairpin structure of the DNA aptamer capturing Ag^+^ ions. (**c**) The structure of Ag-DNA aptamer reversibly changes through binding and dissociating the Ag^+^ ions.

To overcome this challenge, we propose repeatable detection of Ag^+^ ions using a DNA aptamer-linked hydrogel biochemical sensor integrated with a microfluidic heating system (Fig. 1a). The proposed biochemical sensor was divided into three components: a DNA aptamer-linked hydrogel, a micro-heater, and a micro-channel. The DNA aptamers that specifically bind to Ag^+^ ions^16,17^ are cross-linked to the acrylamide hydrogel polymer network to convert the Ag^+^ ions into a volume change of the hydrogel (Fig. 1b). Based on the previous research^17^, the sequence of DNA aptamer that can change their straight structure to the hairpin structure when Ag + ion binding to the DNA aptamer is used (Fig. 1c). The volume change of the DNA aptamer-linked hydrogel is triggered by the folding of the DNA aptamer by capturing the Ag^+^ ions. In more detail, the silver-mediated base pairs (C–Ag(I)–C) in the DNA aptamer are formed between C-residues and change their structure into the hairpin structure (Fig. 1c)^17^. The hairpin structures cause the dragging of the cross-linked hydrogel polymer network, leading to the shrinking of the DNA aptamer-linked hydrogel. For repeatable use of the shrunk DNA aptamer-linked hydrogel, the Ag^+^ ions binding to the DNA aptamer are dissociated by heating and flushing with the microfluidic heating device, enabling the DNA aptamer-linked hydrogel swells to the initial state. Using this mechanism, the proposed DNA aptamer-linked hydrogel biochemical sensor could repeatedly detect Ag^+^ ions because the DNA aptamers in the hydrogel have the capability to capture the Ag^+^ ion again. In this paper, we investigate the properties of Ag^+^ ion responsivity of the DNA aptamer-linked hydrogel and characterize the heating performance of the microfluidic heating device. As a proof of concept, we finally demonstrate the repeatable Ag^+^-ion sensing by the DNA aptamer-linked hydrogel biochemical sensor integrated with the microfluidic heating device.

## Results

### Ag^+^-ion sensing with DNA aptamer-linked hydrogel biochemical sensor

For the DNA aptamer-linked hydrogel biochemical sensor, poly-acrylamide was used as a polymer network material to fix DNA aptamer because of two features of the acrylamide hydrogel: stability in ambient temperature (4–100 °C)^18^ and non-responsiveness to the Ag^+^ ions. We chose Ag^+^-ion DNA aptamer (Fig. 1c) because the Ag^+^ ions are a common indicator to examine the toxicity in the aqueous environment^3^. The ends of the DNA aptamer have been modified with Methacrylic Acid N-Succinimidyl Ester to cross-link with the acrylamide network. To fabricate the DNA aptamer sensor, a pre-gel solution (0.20 g/mL Acrylamide, 0.133% (w/w) *N*,*N*'-methylenebis (acrylamide) as a cross-linker, 0.5% (v/v) Irgacure1173 as a photoinitiator, and Ag^+^-ion DNA aptamer (0, 40, 400 µM in pre-gel solution)) were poured in a poly-dimethylsiloxane (PDMS) molds (Diameter: *d*_m_ = 3 mm, Thickness: *t* = 0.5 mm or Diameter: *d*_m_ = 2 mm, Thickness: *t* = 0.3 mm), then cross-linked by UV irradiation. Finally, the fabricated DNA-aptamer linked hydrogels were placed in the pure water over 3 h for swelling by absorbing the surrounding pure water.

**Figure 2:**
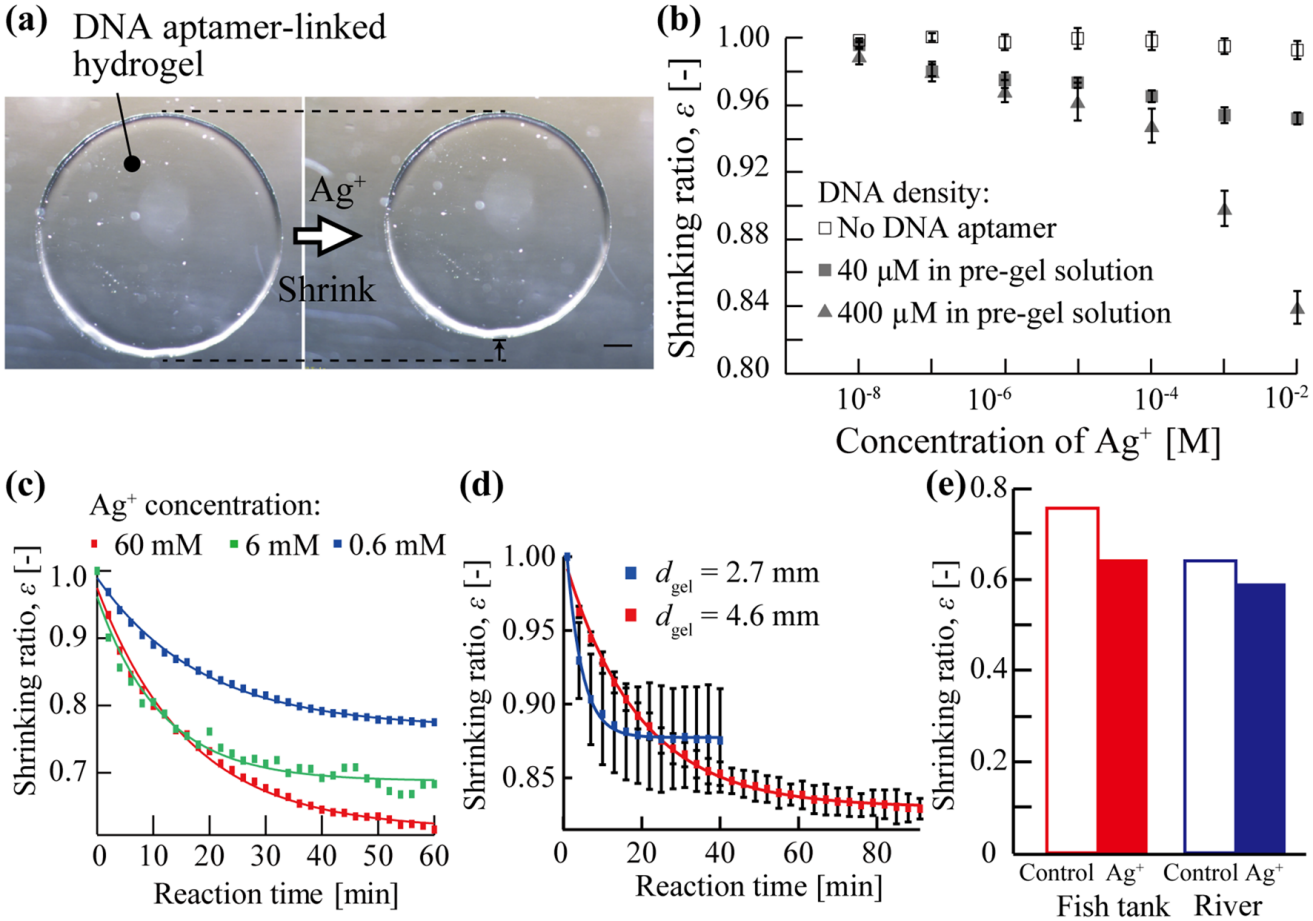
(**a**) The volume change of the DNA aptamer-linked hydrogel when applying the 10 mM Ag^+^ ions. The scale bar is 500 µm. (**b**) The relationship between the shrinking ratio of DNA aptamer-linked hydrogels and the varied Ag^+^ ion concentrations. (**c**) The time variation of the shrinking ratio, *ε*, at different Ag^+^ ion concentrations. (**d**) The time variation of the shrinking ratio, *ε*, for the DNA aptamer-linked hydrogels with different sizes. (**e**) The detection of Ag^+^ ions in the environmental samples.

Firstly, we verified the shrinking behavior of DNA aptamer-linked hydrogel to Ag^+^ ions. The diameters of fabricated DNA aptamer-linked hydrogel, *d*_gel_, were 4.6 mm when we used the mold with 3-mm diameter: *d*_m_ = 3 mm and the thickness: *t* = 0.5 mm (Fig. 2a left). When immersing the DNA aptamer-linked hydrogel into CH_3_COOAg solution for applying the 10 mM Ag^+^ ions, the DNA aptamer-linked hydrogel (DNA aptamer: 400 µM in the pre-gel solution) successfully shrank (Fig. 2a right). On the other hand, the pure acrylamide hydrogel (no DNA aptamer) did not respond to any concentration of Ag^+^ ions (Fig. 2b, white square). These results showed that the shrinking of hydrogel was caused by the DNA aptamer. Thus it is also considered that the DNA aptamer was specifically bound to the Ag^+^ ions and changed their structure to the hairpin structure.

Secondly, the applied Ag^+^-ion concentration was varied from 10^−5^ to 10 mM for observing the influence of the Ag^+^-ion concentration on the shrinking ratio of the DNA aptamer-linked hydrogel. The DNA aptamer-linked hydrogel shrunk isotropically^19^. The shrinking ratio, *ε*, was defined as

1 $$ {\upvarepsilon } = \frac{A\left( t \right)}{{A_{0} }}, $$

where *A*_0_ was an area of DNA aptamer-linked hydrogel before response and *A*(*t*) was an area of DNA aptamer-linked hydrogel at a responding time *t*. The shrinking ratios of the DNA aptamer-linked hydrogels with different DNA aptamer densities (40 µM and 400 µM DNA aptamer in the pre-gel solutions) increased as the Ag^+^-ions concentration increased (Fig. 2b, gray square and gray triangle). The hydrogel with high-density DNA aptamer (400 µM in pre-gel solution) largely changed their volume (*ε* = 0.84 at 10 mM Ag^+^ ions, volume change ratio: 0.77) (Fig. 2b, gray square), while the hydrogel with low-density DNA aptamer (40 µM in pre-gel solution) slightly changed their volume (*ε* = 0. 95 at 10 mM Ag^+^ ions, volume change ratio: 0.93) (Fig. 2b, gray triangle). These results showed that the DNA aptamer-linked hydrogel could be applied to measure the concentration of Ag^+^ ions and the sensitivity of the DNA aptamer-linked hydrogel biochemical sensor can be adjusted according to the amount of DNA aptamer in the hydrogel.

Then, we examined the response speed of the DNA aptamer-linked hydrogel due to the concentration of applied Ag^+^ ions (60 mM, 6 mM, or 0.6 mM). The DNA aptamer-linked hydrogels were gradually changed their volume when applied both 60 mM Ag^+^ ions, 6 mM Ag^+^ ions, and 0.6 mM Ag^+^ ions, and the volume changes converged in ~ 1 h (Fig. 2c red square and blue square, Movie 1). By using a data analysis software (IGOR Pro, WaveMetrics), the plotted shrinking ratios, *ε*, were approximated by an exponential function and a time constant of the exponential function, *τ*, was calculated for comparing the response speed of the DNA aptamer-linked hydrogel sensor. The time constants, *τ*, were approximately the same as 16.3 min (60 mM Ag^+^ ions, Fig. 2c red square), 11.4 (6 mM Ag^+^ ions, Fig. 2c green square), and 18.6 min (0.6 mM Ag^+^ ions, Fig. 2c blue square), while the shrinking ratios were largely different (60 mM Ag^+^ ions: *ε* = 0.62, 6 mM Ag^+^ ions: *ε* = 0.68, 0.6 mM Ag^+^ ions: *ε* = 0.77). These results indicate that the response speed of DNA aptamer-linked hydrogel was not dominantly affected by the Ag^+^ ions concentration.

Then, the influence of the surface-to-volume ratio (*R*_s,v_) was observed by changing the diameter of DNA aptamer-linked hydrogels. DNA aptamer-linked hydrogels with different diameters (*d*_gel_ = 2.7 mm and 4.6 mm) were fabricated with different size of molds (*d*_m_ = 2 mm, *t* = 0.3 mm, *R*_s,v_ = 8.67 and *d*_m_ = 3 mm, *t* = 0.5 mm, *R*_s,v_ = 5.33) and applied with the 10 mM Ag^+^ ions. The diameters of the DNA aptamer-linked hydrogel decreased from 2.7 to 2.5 mm (*ε* = 0.88, Fig. 2d blue square) and from 4.6 to 4.2 mm (*ε* = 0.84, Fig. 2d red), respectively. The time constant of the smaller DNA aptamer-linked hydrogel (*d*_gel_ = 2.7 mm, *R*_s,v_ = 8.67) was faster (*τ* = 3.9 min) than the larger DNA aptamer-linked hydrogel (*d*_gel_ = 4.6 mm, *R*_s,v_ = 5.33, *τ* = 19.0 min). These results showed that the response speed is improved by increasing the surface-to-volume ratio of the DNA aptamer-linked hydrogel without decreasing the shrinking ratio.

Next, the detection of Ag^+^ ions in the environmental samples was observed. The environmental samples were obtained from a fish tank and a river (Supporting Information S1, Fig. S1). The DNA aptamer-linked hydrogels (400 µM DNA aptamer in the pre-gel solutions) were immersed in the environmental samples with or without additional 10 mM Ag^+^ ions over 2 h. The DNA-aptamer linked hydrogel shrunk with *ε* = 0.76 responding to the multi ions in the sample of the fish tank (Fig. 2e, red frame bar). When immersing in the sample of the fish tank with 10 mM Ag^+^, the DNA-aptamer linked hydrogel largely shrunk with *ε* = 0.65 (Fig. 2e, red bar). Similar to the sample of the fish tank, the DNA-aptamer linked hydrogel largely shrunk when immersing the sample of the river with 10 mM Ag^+^ ions (control: *ε* = 0.64, sample with 10 mM Ag^+^: *ε* = 0.59, Fig. 2e blue bars). These results indicated that the DNA-aptamer-linked hydrogel was affected by the multi ions in the surrounding environment. However, the DNA aptamers proposed in this study specifically bind Ag^+^ ions and form the hairpin structure, leading to the large shrink of the DNA-aptamer linked hydrogel (Supporting Information S2, Fig. S2)^15^. In fact, there is a clear difference in shrinkage ratios of the DNA-aptamer linked hydrogel with and without Ag^+^ ions. Therefore, the proposed sensor can detect the Ag^+^ ions in the surrounding environments by measuring the difference of the shrinking behavior.

### Initializing the DNA aptamer-linked hydrogel for repeatable measurement

The dissociation temperature of Ag^+^ ions from DNA aptamer is estimated from the melting temperature, *T*_m_. The DNA aptamer has five A-T base pairs and five C–Ag(I)-C pairs when capturing Ag^+^ ions. In the Wallace method and the previous research^16^, the melting temperature of a DNA duplex increases by 2 °C per A-T base pair (*T*_m, AT_) and 8 °C per C–Ag(I)–C pairs (*T*_m, CAgC_), respectively. Therefore, the melting temperature, *T*_m_, could be calculated as

2 $$ T_{{\text{m}}} = 5 \times T_{{{\text{m}},{\text{AT}}}} + 5 \times T_{{{\text{m}},{\text{CAgC}}}} = 5 \times 2 + 5 \times 8 = 50\;^\circ {\text{C}}{.} $$

**Figure 3:**
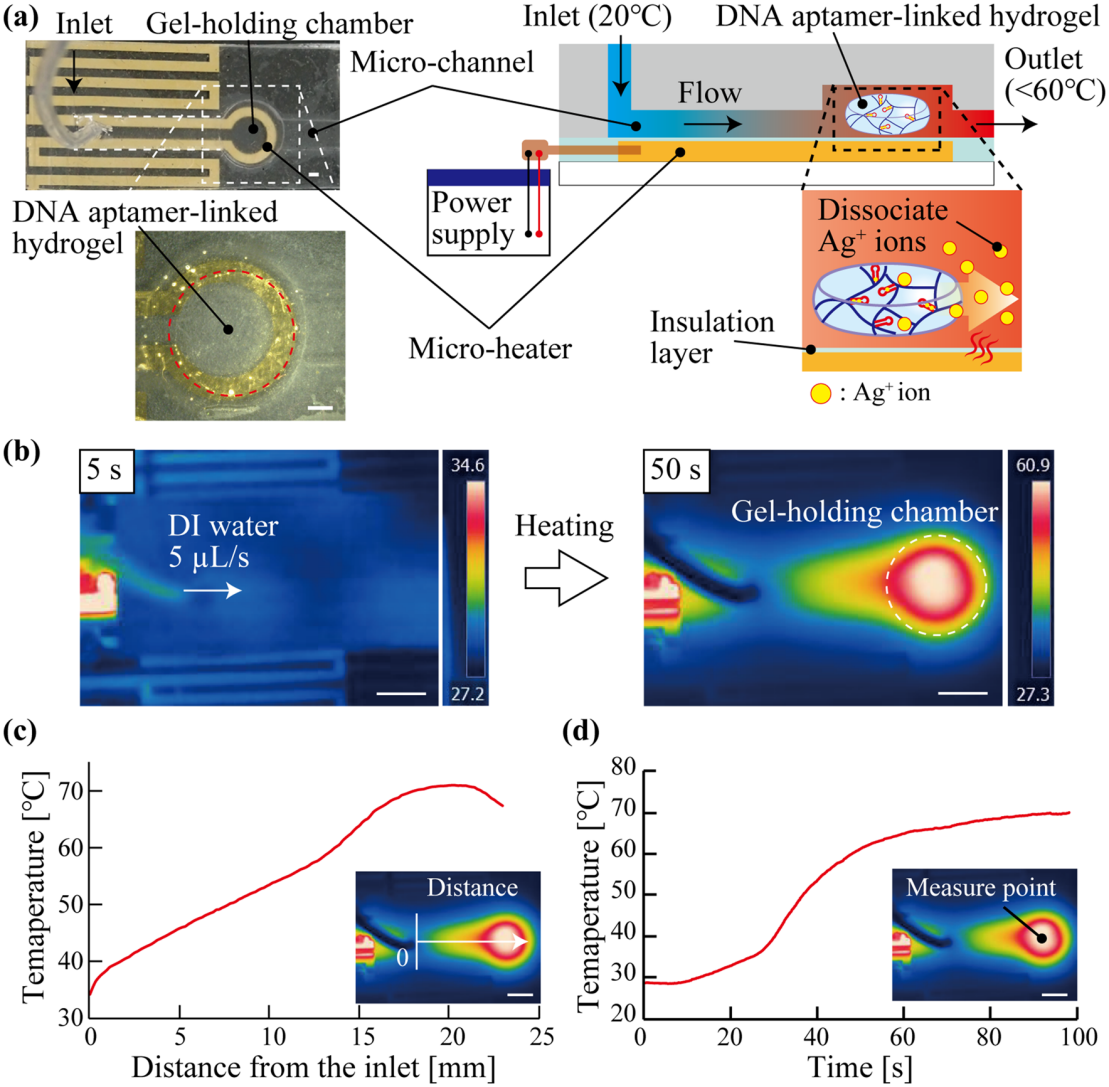
(**a**) Schematic illustration and images of the microfluidic heating device to initialize the DNA aptamer-linked hydrogel for repeated detection. Scale bar is 1 mm. (**b**) Thermographic image of the microfluidic heating device when supplying the DI water and heating by the micro-heater. Scale bar is 5 mm. (**c**) The relationship between the distance from the inlet and the temperature of the micro-channel. Scale bar is 5 mm. (**d**) Time-course temperature measurement of the gel-holding chamber. The scale bar is 5 mm.

To heat the DNA aptamer-linked hydrogel up to the melting temperature for dissociation of the Ag^+^ ions, we fabricated the microfluidic heating device (Fig. 3a left). The microfluidic heating device is divided into two main components: the micro-heater and the micro-channel. The micro-heater made by the patterned Au layer (thickness: ~ 175 nm, width: 1 mm, Fig. S3a) can heat an aqueous solution that flows in the micro-channel by a Joule heat generated by applying electric current (Fig. 3a right). The micro-channel (width: 3 mm, height: 1 mm) has a gel-holding chamber (diameter: 7 mm, height: 1.5 mm) to fix the DNA aptamer-linked hydrogel in the flow (Fig. S3b). The distance from the inlet to the gel-holding chamber was 15 mm.

Then, the characteristics of the fabricated microfluidic heating device were examined. The DI water was supplied to the gel-holding chamber by using the syringe pump at constant flow (5 µL/s) and the supplied DI water was heated by the micro-heater (current: 0.35 A). At the beginning of heating (5 s), the top of the gel-holding chamber was still low temperature (< 30 °C, Fig. 3b left). After 50 s of heating the DI water, the top of the chamber was successfully heated over 50 °C (Fig. 3b right). By measuring the temperature distribution, the temperature of the micro-channel was gradually increased from the inlet to the gel chamber and the temperature at the gel-holding chamber was reached ~ 70 °C (Fig. 3c). In addition, time-course temperature measurement showed that the temperature of the top of the gel chamber was reached 50 °C in approximately 40 s (Fig. 3d). Thereafter, the temperature at the gel-holding chamber was converged at 70 °C. These results indicated that the DNA aptamer-linked hydrogel placed at the gel chamber could be heated over the melting temperature of the DNA aptamer-linked hydrogel, *T*_m_ = 50 °C, by using the fabricated microfluidic heating device.

**Figure 4:**
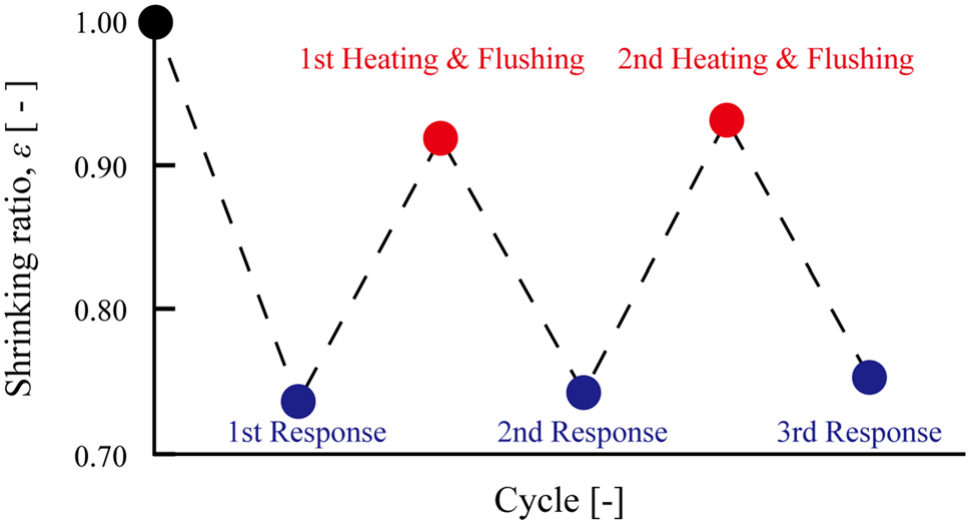
The repeatable detection of Ag^+^ ions by using the DNA aptamer-linked hydrogel biochemical sensor.

Finally, the repeated detection of Ag^+^ ions with the DNA aptamer-linked hydrogel was verified. The DNA aptamer-linked hydrogel (400 µM) shrunk with the shrinking ratio, *ε* = 0.74, on the first response to the 10 mM Ag^+^ ions (Fig. 4 blue plot). Then, the captured Ag^+^ ions with the DNA aptamer-linked hydrogel were dissociated by heating and flushing (Flow of DI water: 1 µL/s, electric current:0.30 A) for 120 min to initialize the sensor. The applied voltage was increased as the temperature of the micro-heater increased. Thus, by using the applied current (0.30 A) and the voltage after converging (6.9 V), the power consumption of the microfluidic device was calculated as 2.07 W. The initialized DNA aptamer-linked hydrogel swelled and the shrinkage ratio, *ε*, recovered to 0.92 (Fig. 4, red plot). The reason why the DNA aptamer-linked hydrogel did not completely swell to the initial state is thought to be due to the plastic deformation of the acrylamide hydrogel network that dragged by folding of the DNA aptamer. Subsequently, the DNA aptamer-linked hydrogel was responded to 10 mM Ag^+^ ions and dissociated by heating and flushing in a repeated manner. The shrinking ratio of the second and third responses were *ε* = 0.74 and *ε* = 0.75 (Fig. 4, blue plots), that were almost the same value of the first response, showing that the responsivity of DNA aptamer-linked hydrogel was maintained even after repeated responses. At the second initializing, the shrinkage ratio of DNA aptamer-linked hydrogel also recovered to *ε* = 0.93, that was also the same value of the first initializing (Fig. 4 red plots). Therefore, these results showed that our proposed DNA aptamer-linked hydrogel biochemical sensor integrated with the microfluidic heating device could detect Ag^+^ ions repeatedly.

## Discussion

Our proposed DNA aptamer-linked hydrogel sensor integrated with the microfluidic heating device showed the capability of the repeated response to Ag^+^ ions. This is the first proposed concept for repeatable measurements of DNA aptamer-linked hydrogel biochemical sensors by initializing with Joule heating. Silver ions are harmful to sensitive aquatic organisms: 1–5 µg/L Ag^+^ ions in an aqueous environment killed sensitive species of aquatic organisms, including representative species of insects, daphnids, amphipods, trout, flounder, and dace; 100–401 µg/L Ag^+^ ions killed various marine animals including scallops, snails, and rainbow trouts; 3000 µg/L Ag^+^ ions killed marine bacteria^3^. The shrinking behavior of the DNA-aptamer linked hydrogel was slightly affected by the multi ions in the environment samples^15,20,21^. However, it was also revealed that the proposed DNA-aptamer linked hydrogel specifically binds to Ag^+^ ions, leading to the large shrinking. The proposed DNA-aptamer linked hydrogel could detect the Ag^+^ ions in the environment samples by comparing the shrinking behavior. Thus, the proposed repeatable biochemical sensor could be applied to long-term water quality monitoring that is necessary to realize a sustainable society.

The initialization of the proposed sensor was demonstrated by low power consumption (approximately 2.07 W) that is an effective characteristic for long-term use. As for the sensing target of the proposed sensor, various types of targets could be acceptable thanks to the variation of DNA aptamer sequences^8,9,22^. In addition, the sensitivity and the response speed of the sensor could be tuned by the density of the DNA aptamer in the hydrogel and the size of the whole hydrogel, respectively, according to the sensing targets.

For practical use of the proposed DNA aptamer-linked biochemical sensor, an automatic volume measurement system that can obtain the diameter of the hydrogel in the microfluidic heating device is necessary because the volume change of the hydrogel was manually measured in this paper. For instance, a Complementary Metal Oxide Semiconductor (CMOS) image sensor has been an attractive candidate because the CMOS image sensor is stable, inexpensive, and easily attached to microfluidic devices to optically monitor the inside condition of the device^23^. Moreover, it could be practically effective that the volume change of the DNA aptamer-linked hydrogel is converted to other information such as visible color change. For example, a photonic colloidal crystal that can convert the volume change into the visible wavelength change has been also expected to be integrated with the DNA aptamer-linked hydrogel because the photonic colloidal crystal can be fabricated in the hydrogel^15,21,24,25^. Regarding the sensing targets, the DNA-aptamer linked hydrogel can respond to the other targets including heavy metal ions by changing the DNA aptamer^8,20,21^. As the basic principle of the binding of the DNA-aptamer to the target substances is the same, the flashing with proposed microfluidic heating devices in this study can apply to other DNA-aptamer linked hydrogels. The melting temperature, *T*_m_, is different by the number of base pairs bound and the binding force between the target substances and the base pairs, but it could be estimated as in Eq. (2). Proposed microfluidic heating device can apply any thermal stimuli by tuning the current, thus the DNA-aptamer linked hydrogel with different DNA aptamers could also detect the target substances repeatedly. With those improvements, we believe that the repeated detection of chemical substances from our body or in environments could be fully automated with our proposed DNA-aptamer-based hydrogel sensor systems.

## Conclusions

We proposed the repeatable detection of Ag^+^ ions by using the DNA aptamer-linked hydrogel biochemical sensor integrated with the microfluidic heating system. The DNA aptamer-linked hydrogel biochemical sensor can detect the wide range of Ag^+^-ion concentrations (10^−5^–10 mM) including the toxic range for various aquatic organisms. Our proposed DNA aptamer-linked hydrogel biochemical sensor could detect the Ag^+^ ions three times by heating and flushing through the microfluidic heating device. The DNA-aptamer linked hydrogel is stable against a wide range of temperatures (4–100 °C), so our proposed biochemical sensor is thus expected to use for long-term monitoring with high stability in ambient temperature and low power consumption. In the future, we also expected that our proposed DNA aptamer-linked hydrogel biochemical sensor could be applied to detect various targets from our body or in environments by adjusting the DNA aptamer sequence.

## Methods

### Fabrication of DNA aptamer-linked hydrogel

The pre-gel solution (0.20 g/mL Acrylamide (Wako, 012-00762) + 0.133% (w/w) *N*,*N*'-methylenebis (acrylamide) (Cross-linker, Wako, 018-03282) + 0.5% (v/v) Irgacure 1173 (Photoinitiator, BASF, 30472687) + Ag^+^-ion DNA aptamer (Aptamer sequence: CH_2_=C(CH_3_)–C(=O)–NH-5′-CTCTCTTCTCAAA-AAACACAACACAC-3′-NH–C(=O)–C(CH_3_)=CH_2_, 0, 40, 400 µM in pre-gel solution, Synthesized by Tsukuba oligo service) was used for fabricating the DNA aptamer-linked hydrogel. The pre-gel solutions were poured into a PDMS mold (Diameter: *d*_m_ = 3 mm, Thickness: *t* = 0.5 mm or Diameter: *d*_m_ = 2 mm, Thickness: *t* = 0.3 mm, base material: curing agent = 10:1, Toray, SILPOT 184) and the PDMS mold was covered by a glass plates (22 × 22 mm, No.1, Matsunami). Then, the pre-gel solution in the PDMS mold was cross-linked by UV irradiation. To replace the electrolyte in the DNA solution with DI water, the fabricated DNA aptamer-linked hydrogel was immersed in 8 mL of DI water for 3 h and washed three times.

### Fabrication of microfluidic heating device

The microfluidic heating device was divided into the micro-heater part and the micro-channel part. The micro-heater part was fabricated by following process (the design details were described in Supporting Information). Chromium (Cr: ~ 5 nm) and gold (Au: ~ 175 nm) layers were deposited on a glass substrate (S9111, Matsunami) by a vacuum evaporator (VE-2012, Vacuum device) as an adhesion layer and a micro-heater wire layer, respectively. For micro-patterning of the micro-heater pattern, the Cr/Au deposited substrate was pre-baked at 100 °C for 2 min, followed by spin-coating of a positive photoresist (OFPR-800-20CP, Tokyo ohka kogyo) at 2000 rpm, and then baked at 100 °C for 4 min. The UV was irradiated to the photoresist-coated substrate through a photomask using a mask aligner (EMA-400, Union). The photoresist layer was developed by NMD-3 (2.38% tetramethylammonium hydroxide, Tokyo ohka kogyo) after the UV irradiation, and then the residual photoresist was removed by O_2_ plasma ashing (SEDE-P, meiwafosis) for 10 s. The exposed Cr/Au were etched by an Au etchant (0.2 M KI + 0.033 M Iodine solution; KI: 164-03972, Wako; Iodine Solution: 094-01705, Wako) and a Cr etchant (Kato chemicals), respectively. After being washed by DI water twice, the photoresist layer was removed by acetone (013-00356, Wako), followed by a rinse with ethanol (057-00456, Wako) and by O_2_ plasma cleaning for 30 s. For wiring to the micro-heater part, Cu wires (2UEW0.26 mm, Kyowa harmonet) were connected to the Au pattern through a conductive paste (No Solder, Elephantech). Finally, a Cytop (CTL-809 M, AGC chemicals) was spin-coated on the Au-etched substrate with 1000 rpm for insulation and then baked at 180 °C for 2 h.

Next, for the micro-channel fabrication (the detailed design was described in Supporting Information.), the PDMS (base material: curing agent = 10:1) was poured into an acryl mold and cured by heating at 75 °C for 2 h. The fabricated micro-cannel part was placed on the heater part after an inlet was opened by a punch biopsy (*φ*1 mm, BP-10F, Kai medical). The inlet was connected to a syringe pump (KD Scientific, LEGATO 180) via a tefzel tube (VICI, 1/16″ × 0.5 ETFE).

### Observation of DNA aptamer-linked hydrogel

The DNA aptamer-linked hydrogel was observed by an inverted phase-contrast microscope (OLYMPUS, IX73P1-22FL/PH). For observing the response to Ag^+^ ions, the DNA aptamer-linked hydrogel was soaked into 1 µM–10 mM silver acetate solutions (CH_3_COOAg) for 120 min. For comparing the responding speed, by using a data analysis software (IGOR Pro, WaveMetrics), the plotted shrinking ratios, *ε*, were approximated by an exponential function and a time constant of the exponential function, *τ*, was calculated for comparing the response speed of the DNA aptamer-linked hydrogel sensor.

For detection of Ag^+^ ions in the environmental conditions, the environmental samples were obtained from the fish tank and the river. The DNA-aptamer linked hydrogel (DNA aptamer: 400 µM in the pre-gel solution) was immersed in the environmental samples with or without 10 mM Ag^+^.

### Investigating the characteristics of the microfluidic heating device

To investigate the micro-heater function of the microfluidic heating device, an electric current (0.35 A) was applied to the micro-heater part by a power source (PS40-20A, TEXIO). The DI water was induced at 5 µL/s in the micro-channel part by the syringe pump. The temperature of the micro-channel was measured by a thermography camera (ETS320, FLIR).

### Repeatable detection of Ag^+^ ions using the DNA aptamer-linked hydrogel

For repeatable detection using the DNA aptamer-linked hydrogel, followed three processes were repeated three times. Firstly, the DNA aptamer-linked hydrogel (DNA aptamer: 400 µM in the pre-gel solution) was responded to Ag^+^ ions by soaking in 10 mM CH_3_COOAg solution for 120 min. Then, the DNA aptamer-linked hydrogel was heated and flushed by the microfluidic heating system (Flow of DI water: 1 µL/s, electric current:0.30 A) for 120 min to dissociate Ag^+^ ions. Finally, the DNA aptamer-linked hydrogel was cooled in the DI water (20 °C) for 120 min.

## Supplementary Information

Supplementary Video 1.

Supplementary Figures.
