# Supplementary material for: Repeatable detection of Ag+ ions using a DNA aptamer-linked hydrogel biochemical sensor integrated with microfluidic heating system
Source: Sci Rep. 2022 Jun 11;12:9692. doi: 10.1038/s41598-022-13970-z (PMC9188593; doi:10.1038/s41598-022-13970-z)
Supplement: Supplementary file 2 — Supplementary Figures. [file 41598_2022_13970_MOESM2_ESM.pdf]

Supporting Information

Repeatable detection of Ag^+ ions using a DNA aptamer-linked hydrogel biochemical sensor integrated with microfluidic heating system

Koki Yoshida[1‡], Tomoki Hayashi[1‡], Masahiro Takinoue[3], Hiroaki Onoe[1,2]*

[1]Graduate School of Integrated Design Engineering, Keio University 3-14-1 Hiyoshi, Kohoku-Ku, Yokohama, 223-8522, Japan.

[2]Department of Mechanical Engineering, Faculty of Science and Technology, Keio University 3-14-1 Hiyoshi, Kohoku-Ku, Yokohama, 223-8522, Japan.

[3]Department of Computer Science, School of Computing, Tokyo Institute of Technology, 4259 Nagatsutacho, Midori-Ku, Yokohama, 226-8503, Japan.

Table of Contents

S1. Environmental samples (Figure S1)
S2. The specificity of the DNA-aptamer linked hydrogel (Figure S2)
S3. Design of the microfluidic heating device (Figure S3)
S4. References of the Supporting Information

S1. Environmental samples

For the detection of Ag^{+} ions in the environmental samples, the environmental samples were obtained from a fish tank and a river. We kept a sturgeon in the laboratory, the sample was obtained after 3 days from cleaning the tank (Figure S1 (a)). The sample of the river was obtained from the Yagami River that flows near the Keio University Yagami Campus (Figure S1 (b)).

(a) **(b)**

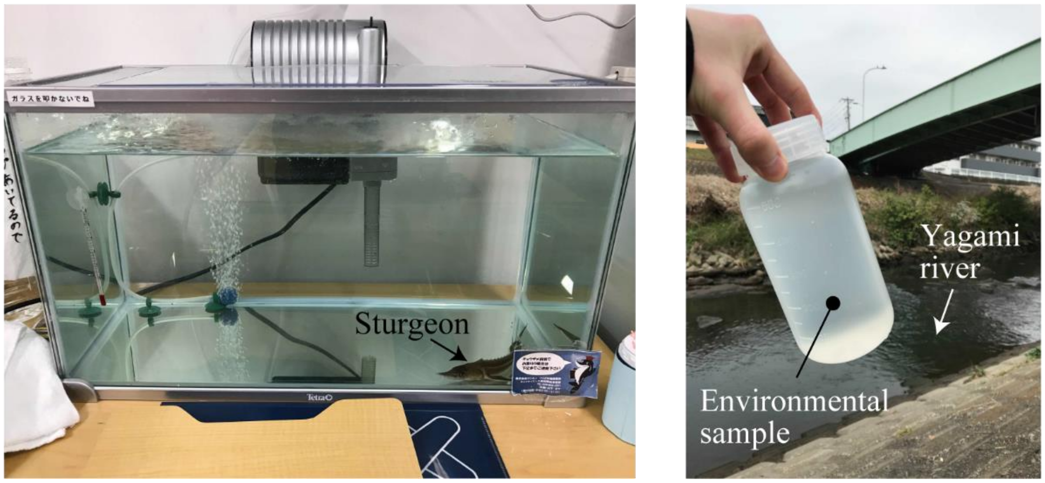

Figure S1. (a) The fish tank. (b) The river.

S2. The specificity of the DNA-aptamer linked hydrogel

For confirming the specificity of the proposed DNA-aptamer linked hydrogel, the shrinking behaviors of the DNA-aptamer linked hydrogels were observed when applied 0.1 µM CH_3COOAg (Ag^+), 1 µM CH_3COOAg (Ag^+), 1 µM $MgCl_2$ (Mg^{2+}), and 1 µM $CaCl_2$ (Ca^{2+}). When applied the 0.1µM Ag^+ and 1 µM Ag^+, the DNA-aptamer linked hydrogel shrunk with the shrinking ratio, $\varepsilon = 0.99$ and 0.97, respectively (**Figure S2 red bars**). On the other hand, the DNA-aptamer linked hydrogel swell by applying the 1 µM Mg^{2+} and 1 µM Ca^{2+}, and each shrinking rations were $\varepsilon = 1.06$ (Mg^{2+}, **Figure S2 blue bar**) and $\varepsilon = 1.06$ (Ca^{2+}, **Figure S2 green bar**). Those results indicate that the interaction between DNA-aptamers and ions slightly affects the swelling and shrinking behavior of the DNA-aptamer linked hydrogel as the previous research[1,2,3], however, it was also revealed that the DNA-aptamer specifically binds to Ag^+ ions.

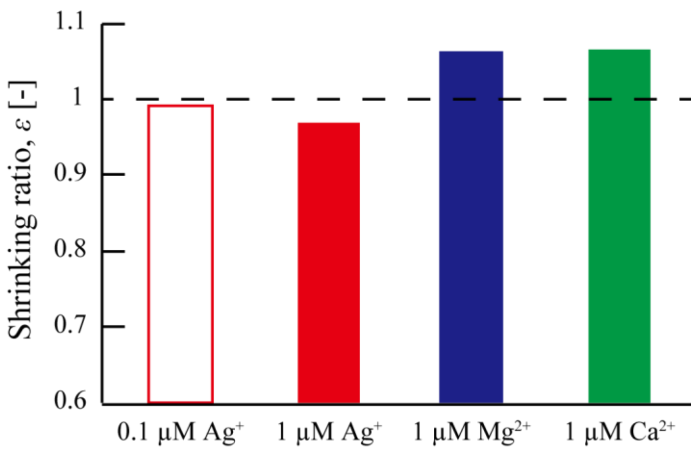

Figure S2. The specificity of the DNA-aptamer linked hydrogel.

S3. Design of the microfluidic heating device

The micro-heater wire is routed along the inlet side of the microfluidic channel to the gel-holding chamber section (The width of the micro-fluidic channel part: 1 mm, the gel-holding chamber part: 0.9 mm, **Figure S3 (a)**). The radius of the gel-holding chamber part is 2.05 mm. For connecting to the energy supply, the micro-heater wire has two connection parts (3.0 mm square). The Cu wires are connected to these connection parts through the conductive paste.

The width of the micro-channel is 3.0 mm and the diameter of the gel-holding chamber is 7.0 mm. The height of the micro-channel and the gel-holding chamber is 1.0 mm and 1.5 mm, respectively (**Figure S3 (b)**).

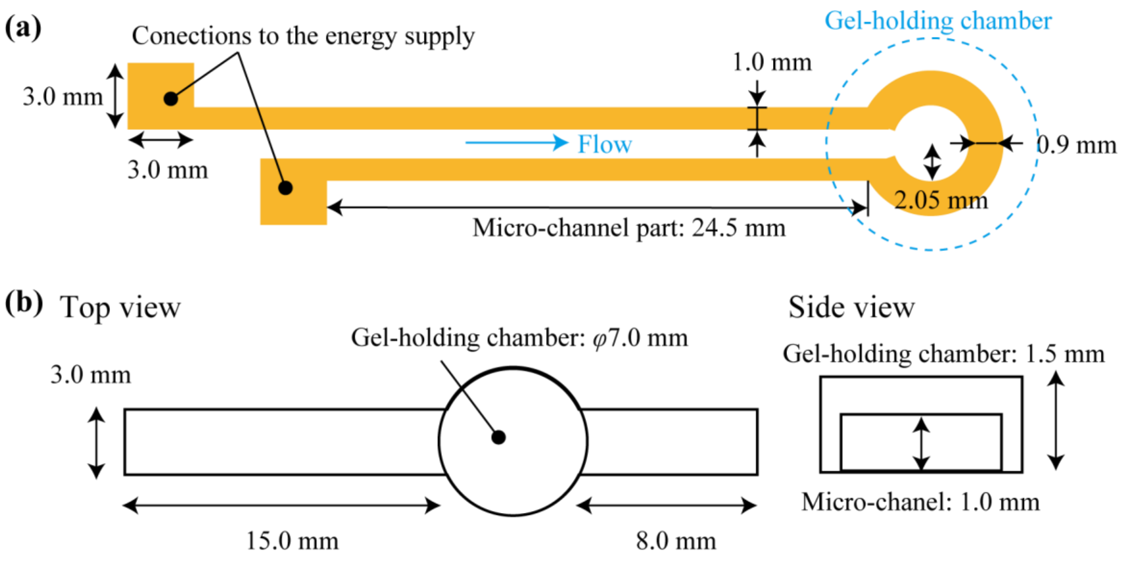

Figure S3. (a) The design of the micro-heater part. (b) The design of the micro-channel part.

S4. References of the Supporting Information

1. Ye, B. *et al.* Colorimetric logic response based on aptamer functionalized colloidal crystal hydrogels. *Nanoscale* **7**, 7565–7568 (2015).

2. Wang, C., Li, F., Bi, Y. & Guo, W. Reversible Modulation of 2D Photonic Crystals with a Responsive Shape-Memory DNA Hydrogel Film. *Adv. Mater. Interfaces* **6**, 1–8 (2019).

3. Ye, B. F. *et al.* Colorimetric photonic hydrogel aptasensor for the screening of heavy metal ions. *Nanoscale* **4**, 5998–6003 (2012).
